# Phoenix criteria for sepsis: are these enough to guide a clinician?

**DOI:** 10.1007/s00431-024-05767-1

**Published:** 2024-09-11

**Authors:** Isadora Rodriguez, Akash Deep

**Affiliations:** 1 Paediatric Intensive Care Unit, King’s College Hospital NHS Foundation Trust, Denmark Hill, London, UK; 2 Department of Women and Children’s Health, School of Life Course Sciences, King’s College London, London, UK

**Keywords:** Sepsis, Septic shock; Phoenix criteria; Children; Intensive care

## Abstract

Sepsis is the leading cause of mortality in children worldwide. There is a paucity of data on the criteria used to define sepsis and septic shock and predict mortality. Schlapbach et al. published Phoenix criteria to define sepsis in JAMA in 2024. Previously, paediatricians have used systemic inflammatory response syndrome (SIRS) criteria, but these criteria lack sensitivity and specificity. This group recommends that sepsis in children be identified by a Phoenix Sepsis Score of at least 2 points in children with suspected infection, which indicates potentially life-threatening dysfunction of the respiratory, cardiovascular, coagulation, and/or neurological systems. Though included in the 8-point criteria, important criteria like renal and liver are missing from the main criteria. We remain worried about the way these criteria got excluded from the main criteria. Therefore, in this brief report, whilst commending the authors for this stelar task, we highlight the main pitfalls in these criteria especially the renal, neurologic, and liver criteria. These criteria have been shown to be independently associated with outcomes, and we recommend that in the future iterations of the criteria, renal and liver criteria should be defined according to latest definitions and the task force consider utilizing latest criteria for each organ system involved within the formulated criteria.

*Conclusion*: In conclusion, Phoenix criteria are a step in the right direction to define life-threatening organ dysfunction in sepsis, but clinicians need to be mindful that diagnosis/treatment of less severe sepsis should not be delayed if these criteria are not met. Therefore, local early detection and management tools for sepsis should be followed.

**Table:** 

**What is Known:** *• There has always been a quest for a definition for pediatric sepsis. There are limitations to the previous pediatric sepsis criteria which were published in 2005 by the International Pediatric Sepsis Consensus Conference (IPSCC). IPSCC defines sepsis as a suspected or confirmed infection in the presence of systemic inflammatory response syndrome (SIRS). These new Phoenix Pediatric Sepsis (PPS) criteria for sepsis and septic shock are intended to identify children with life-threatening organ dysfunction due to infection, and the score was developed based on a very large pediatric dataset.*	
**What is New:** *• Though the intention of Phoenix criteria is to help identify children with life threatening organ dysfunction, unfortunately the crietria will miss signs of early sepis. In this manuscript, we point out some of the drawbacks of these criteria which need to be borne in mind while applying these criteria.*

**What is Known:**

*• There has always been a quest for a definition for pediatric sepsis. There are limitations to the previous pediatric sepsis criteria which were published in 2005 by the International Pediatric Sepsis Consensus Conference (IPSCC). IPSCC defines sepsis as a suspected or confirmed infection in the presence of systemic inflammatory response syndrome (SIRS). These new Phoenix Pediatric Sepsis (PPS) criteria for sepsis and septic shock are intended to identify children with life-threatening organ dysfunction due to infection, and the score was developed based on a very large pediatric dataset.*

**What is New:**

*• Though the intention of Phoenix criteria is to help identify children with life threatening organ dysfunction, unfortunately the crietria will miss signs of early sepis. In this manuscript, we point out some of the drawbacks of these criteria which need to be borne in mind while applying these criteria.*

Sepsis is up until today the leading cause of death in children; however, defining sepsis has been a great challenge especially in children. It was with great interest that we read the manuscript “International Consensus Criteria for Pediatric Sepsis and Septic Shock” by Schlapbach et al. [1].

Discussing sepsis in paediatric settings is not an easy feature specially with the amount of data that this team was able to acquire. The authors were able to capture a large, diverse, and multiethnic population with the aim to achieve a scoring system that would be applicable in both low-middle and high-resource settings [2].

Though these criteria have been well received, through this brief report, we would like to highlight some of the shortcomings of these criteria. Although some of the criteria chosen were selected based on the best individual performance, they seem to show late clinical alterations and only highlight the importance of having a diagnostic tool that could be used in the early onset of suspected sepsis, when the possibility of changing the outcome is higher. At the moment, it is unclear when in the trajectory of sepsis progressing to septic shock, should these criteria be used.

In the case of neurological dysfunction, authors have used pupillary reaction to light as one of the criteria. In most cases, the lack of pupillary reaction can happen as a sign of irreversible neurological dysfunction that is associated with poor outcome [3]. Considering this usually takes place at a later/terminal stage, using other tools to identify children with neurological dysfunction, such as the AVPU Scale could still maintain the purpose of being easily accessible and might identify earlier changes [4–6]. Although we understand that the Phoenix sepsis criteria is not meant to be an early detection tool, maintaining such late detection criteria might make this scoring system less meaningful for its daily use.

In addition, renal dysfunction is not clearly addressed in the main 4-point criteria whereas there are clear indicators that renal dysfunction can happen in any stage of sepsis and septic shock as reviewed by the ADQI (Acute Disease Quality Initiative) group where it was discussed that sepsis-associated acute kidney injury (SA-AKI) is common in critically ill patients and is strongly associated with adverse outcomes, including an increased risk of chronic kidney disease, cardiovascular events, and death [7]. The authors have used a fixed value of creatinine in their criteria which brings with it the challenges in malnourished children and children with liver disease especially those with chronic liver failure and importantly does not consider the time-line of a delta change in creatinine value which is what is used in the current KDIGO (Kidney Disease Improving Global Outcomes) guidelines. The authors seem to have used creatinine only in the AKI diagnostic criteria excluding urine output which, in fact, has been shown to be associated more closely with mortality than creatinine alone. In addition, urine output has been traditionally used as a marker of tissue perfusion, particularly related to microcirculation, and provides valuable information when there is ongoing sepsis/septic shock. Therefore it is unclear how the authors weighted AKI in the final score based on a single creatinine value without demonstrating a delta change in baseline value. In addition, the definition of AKI has undergone an evolution over the years with changing weightage given to serum creatinine changes.

Regarding liver dysfunction caused by sepsis, its presence has been described as a powerful and independent predictor of mortality in children which could demonstrate the importance of adding this assessment to the criteria [8]. With regard to sepsis, the liver participates in two main mechanisms: the first is the regulation of immune defense via hepatic sinusoids by ultimately producing acute phase proteins. It also acts on bacterial and endotoxin clearance from the bloodstream by producing neutrophil extracellular traps, which essentially mimic the effect of a net and block bacteria passage [9]. This, however, reduces blood flow and could play a role in the impaired hepatic microcirculation seen in sepsis nature. The second is direct liver injury: The process can begin with hypoxic hepatitis, with the increase of alanine aminotransferase and aspartate aminotransferase, that in one third of the cases can progress to cholestatic hepatic dysfunction [9]. Alternatively, some, but not all, patients can first present with sepsis-induced cholestasis from ductular or hepatocellular levels, without necessarily a mechanical obstruction of the biliary tree. These changes can be a result from the inflammatory state generated by sepsis, and serum bilirubin levels are a key marker of this type of dysfunction nature [9]. The authors have used serum bilirubin and ALT cut-offs in the 8-point criteria. As mentioned, not every patient will have raised serum bilirubin at presentation or in the immediate period post PICU admission.

We are surprised by the inclusion of coagulation parameters in the main criteria. Besides the difficulty in getting D-dimers and INR in all healthcare settings associated with costs for these tests, interpretation in the setting of liver disease becomes an issue.

Lastly, we agree that there should be cardiovascular dysfunction to define septic shock; however, having such a wide range of values for the lactate threshold can misguide primary carers and delay recognition. It is also important to remember that not all patients will present with a raised lactate, so assessing other markers of hypoperfusion is important. There was no mention of fluid resuscitation in the criteria. Considering that this is a treatment that is widely available in all income settings and the lack of response to it can indicate the severity of the disease, it would be important to consider this variable [10]. Fluid refractory septic shock denotes increased severity or paucity of resources (such as settings with no intensive care unit available) which implies the need for vasoactive drugs and admission to paediatric intensive care units where available [10].

In conclusion, Phoenix sepsis criteria are a step in the right direction in the field of defining sepsis; however, we hope that in the future iteration, the task force consider utilizing the latest criteria for each organ system involved within the formulated criteria. Can more work be done to have sepsis defining criteria at presentation which can aid the healthcare professionals in not-missing this life-threatening condition and instituting timely treatment to improve short- and long-term outcomes?

## Data Availability

No datasets were generated or analysed during the current study.
